# Global, regional, and national burdens of Alzheimer's disease and other forms of dementia in the elderly population from 1999 to 2019: A trend analysis based on the Global Burden of Disease Study 2019

**DOI:** 10.1002/ibra.12181

**Published:** 2024-09-22

**Authors:** Mengdan Su, Tianhong Wang, Congcong Zou, Keyu Cao, Fei Liu

**Affiliations:** ^1^ Department of Anesthesiology, West China Hospital Sichuan University Chengdu China; ^2^ Department of Nursing, West China Hospital Sichuan University Chengdu China; ^3^ West China School of Nursing Sichuan University Chengdu China

**Keywords:** Alzheimer's disease, dementias, disability‐adjusted life years, elderly population, global burden of disease

## Abstract

Dementia represents a significant health issue, afflicting both patients and their families. To assess the global trends in the incidence, prevalence, mortality, and disability‐adjusted life years (DALYs) of Alzheimer's disease (AD) and other dementias in the elderly population, the Global Burden of Disease Study (1999−2019) was used. The average annual percentage change (AAPC) was estimated using linear regression. Stratified analysis of the global trends by age, sex, region, national level, and social development index (SDI) were also performed. The global incidence of AD and other dementias increased from 507.96 per 100,000 in 1990 to 569.39 per 100,000 in 2019, showing a significant increase in this period. In males, the incidence increased from 387.56 per 100,000 population in 1990 to 462.40 per 100,000 in 2019 (AAPC = 0.61), whereas females experienced a slower rise (AAPC = 0.31) and had a higher incidence in 2019 (662.93 per 100,000 population). The most significant increase was observed in individuals aged 60−64 and those in the middle‐SDI quintile. Regionally, the high‐income Asia Pacific had the highest incidence (890.01 per 100,000 population) and DALYs (3043.86 per 100,000) in AD and other dementias in 2019. As for national trends, Japan had the most pronounced increase in the incidence and DALYs of AD and other dementias during the 1990−2019 period. These findings highlight the growing burden of dementias on life expectancy at a population level, which is significant for healthcare professionals and decision‐makers to conduct the ongoing debate on the policy of AD and other dementias.

## Graphical abstract

This study investigated the global trends in the incidence, prevalence, mortality, and disability‐adjusted life years (DALYs) of Alzheimer's disease (AD) and other dementias in the elderly population (60−89 years) from 1990 to 2019. The global incidence of AD and other dementias increased from 508.0 per 100,000 in 1990 to 569.4 per 100,000 in 2019. Moreover, the global prevalence ascended from 3463.1 per 100,000 in 1990 to 4081.4 per 100,000 in 2019. The global mortality attributed to AD and other dementias elevated from 88.0 per 100,000 in 1990 to 106.2 per 100,000 in 2019. The DALYs of AD and other dementias increased from 1657.4 per 100,000 in 1990 to 1935.9 per 100,000 in 2019.

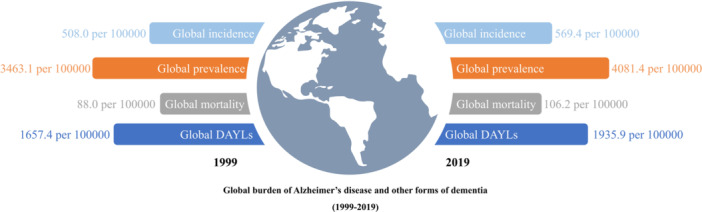

## 1 INTRODUCTION

Dementia is a syndrome, with Alzheimer's disease (AD) being the most common type^1^ and recognized by the World Health Organization (WHO) as a global public health priority.^2^ Characterized by progressive cognitive impairment, dementia affects daily activities and is a significant cause of dependence, disability, and mortality.^3^ This condition is a profoundly devastating disorder that affects not only the patients but also their caregivers, families, and society at large.^4^ Against the background of an aging society, the burden of dementia is hitting high notes.

Aging is a global phenomenon that is affecting countries worldwide.^5^ The number of individuals aged 65 or older is expected to grow from 420 million in 2000 to nearly 1 billion by 2030, with the proportion increasing by 7%–12%.^6^ Dementia primarily affects elderly individuals, and the occurrence and prevalence increase as age advances.^7^ According to reports, the worldwide prevalence of dementia has risen significantly in recent years. In 2016, the total number of affected individuals reached 43.8 million, marking a substantial 117% increase compared to the 20.3 million recorded in 1990.^8^ Projections indicate that by 2050, the global population living with AD and other forms of dementia is expected to reach a staggering 152 million.^9^ These data highlight the necessity to assess the global impacts of AD and other dementias in the elderly population, which is beneficial to developing effective intervention strategies.

The Global Burden of Disease Study (GBD) 2019, by integrating various data sources such as literature, medical institution records, and publicly available databases, is used to analyze epidemiological data for 204 countries and regions. This comprehensive analysis included patients of both sexes and 369 diseases.^8^ Based on the GBD project, this study aims to assess the global trends (1999−2019) in the incidence, prevalence, mortality, and disability‐adjusted life years (DALYs) of AD and other dementias in the elderly population. In addition, the trends at the regional and national levels were also evaluated. DALYs represent the number of healthy years lost due to morbidity and are widely considered the standard indicator of disease burden.^10^ Our findings associated with the trends of AD and other dementias might provide insights into the potential risk of increasing dementias on healthy lifespan at a population scale, which is significant for healthcare professionals and decision‐makers to inform the ongoing debate on the policy of AD and other dementias.

## 2 MATERIALS AND METHODS

### 2.1 Study population and data collection

Leveraging the GBD 2019, we derived multi‐wave cross‐sectional data from the Global Health Data Exchange (GHDx, https://ghdx.healthdata.org/), which investigates the prevalence of 369 diseases and injuries across 204 countries and territories between 1990 and 2019, such as AD and other dementias. The total number of included populations is 5,349,847,687 in 1990, 6,075,911,237 in 1999, 6,903,480,102 in 2009, and 7,737,464,623 in 2019.

Dementia is a chronic neurological disorder characterized by progressive, degenerative cognitive dysfunctions that disrupt daily functioning. The data were collected by referring to the criteria outlined in the Diagnostic and Statistical Manual of Mental Disorders (DSM) editions III, IV, or V or the International Classification of Diseases (ICD) case definitions in GBD. Data on AD and other dementias were obtained from both sexes, males and females, in four age groups (60−64, 65−69, 60−79, and 60−89 years), according to the 21 regional clusters of countries defined in the GBD project and grouped based on geographical proximity and epidemiological similarities.^8^ Senescence represents the process of aging in organisms. It is characterized by a gradual decrease in physiological functions and increased vulnerability to disease and death. The WHO defines senescence as age over 60 years. In this work, we defined the study population as those aged between 60 and 89 years. Based on the GBD results, we employed age subgroups of 60−64, 65−69, 60−79, and 60−89 years to delineate in more detail aging growth and popular understanding of the late life phase.

To evaluate the impact of social and economic factors on AD and other dementias, the social development index (SDI) was computed for each country in the GBD 2019 study. This index is a composite measure reflecting the impact of social and economic factors on health outcomes. It is determined by the geometric mean of normalized indices, including the total fertility rate for individuals under 25 years, average educational attainment for those aged 15 and above, and income per capita, adjusted for distribution delays.^8^ The SDI ranges from 0 (indicating minimal education, lowest income, and highest fertility) to 1 and is categorized into five levels: low, low‐middle, middle, high‐middle, and high.

Data on incident and prevalent cases, deaths, DALYs, incidence, and prevalence rates were directly collected from the GBD 2019 study, with all rates presented per 100,000 population. The 95% uncertainty intervals (UIs) were determined using the 25th and 975th values from the 1000 estimates generated by the GBD algorithm. The methodology of GBD 2019, including these calculations, has been described in detail in prior publications.^8^, ^11^, ^12^

The Ethics Committee of the West China Hospital ruled that approval was not required for this study, as it was sourced from publicly accessible data. The study adhered to the Guidelines for Accurate and Transparent Health Estimates Reporting for cross‐sectional studies.

### 2.2 Statistical analysis

First, this study aimed to determine the global trends in the incidence, prevalence, mortality, and DALYs of AD and other dementias. We estimated the age‐specific rates and average annual percentage changes (AAPCs) using a linear regression model. The AAPC for a given fixed interval is derived by taking a weighted average of the slope coefficients from the joinpoint regression model applied to the data. In this calculation, the weights are determined by the duration of each segment within the interval. The culmination of this process involves converting the weighted average of these slope coefficients into an annual percentage rate of change. The rates were log‐transformed as the dependent variable, and years were independent log‐transformed as the independent variable. The AAPC is a consolidated metric used to quantify trends over a set time frame. It is calculated as the weighted mean of the annual percentage change (APC), enabling the representation of average APC across a period of years with a single value. The APC was estimated by the geometrically weighted average of the different APC in the regression model. Therefore, the estimate of the AAPC reported that the annual percentages changed to some extent, such as increasing, decreasing, or remaining stable. The trends in the rates were presented as AAPC values and their 95% confidence interval (CI). To evaluate the changes in various periods, we calculated AAPCs during four intervals, including 1990−1999, 2000−2009, 2010−2019, and 1990−2019.

Second, we aimed to capture the year with the most significant changes in trends. Joinpoint regression analysis was used to discern trends in the data over time. This method involves fitting the most straightforward model to the data by connecting various linear segments on a logarithmic scale. In this avenue, segments called “joinpoints” are identified, with the simplest model (0 joinpoints) represented by a straight line. The inclusion of additional joinpoints is evaluated using the Monte Carlo permutation method. The weighted Bayesian information criterion method was applied in Joinpoint software to determine the most appropriate model.

Third, the global trend was stratified by age, sex, and SDI group, and regional and national trends were also calculated. Similarly, we calculated AAPCs for each group with CIs and reported and interpreted the GBD results with cases, rates, and UIs, alongside *p* values.^13^ *p* < 0.05 is considered statistically significant. All the statistical analyses were done using R version 4.2.1 and the Joinpoint Regression Program (4.9.1.0).

## 3 RESULTS

### 3.1 Global trends in the incidence of AD and other dementias

**Figure 1:**
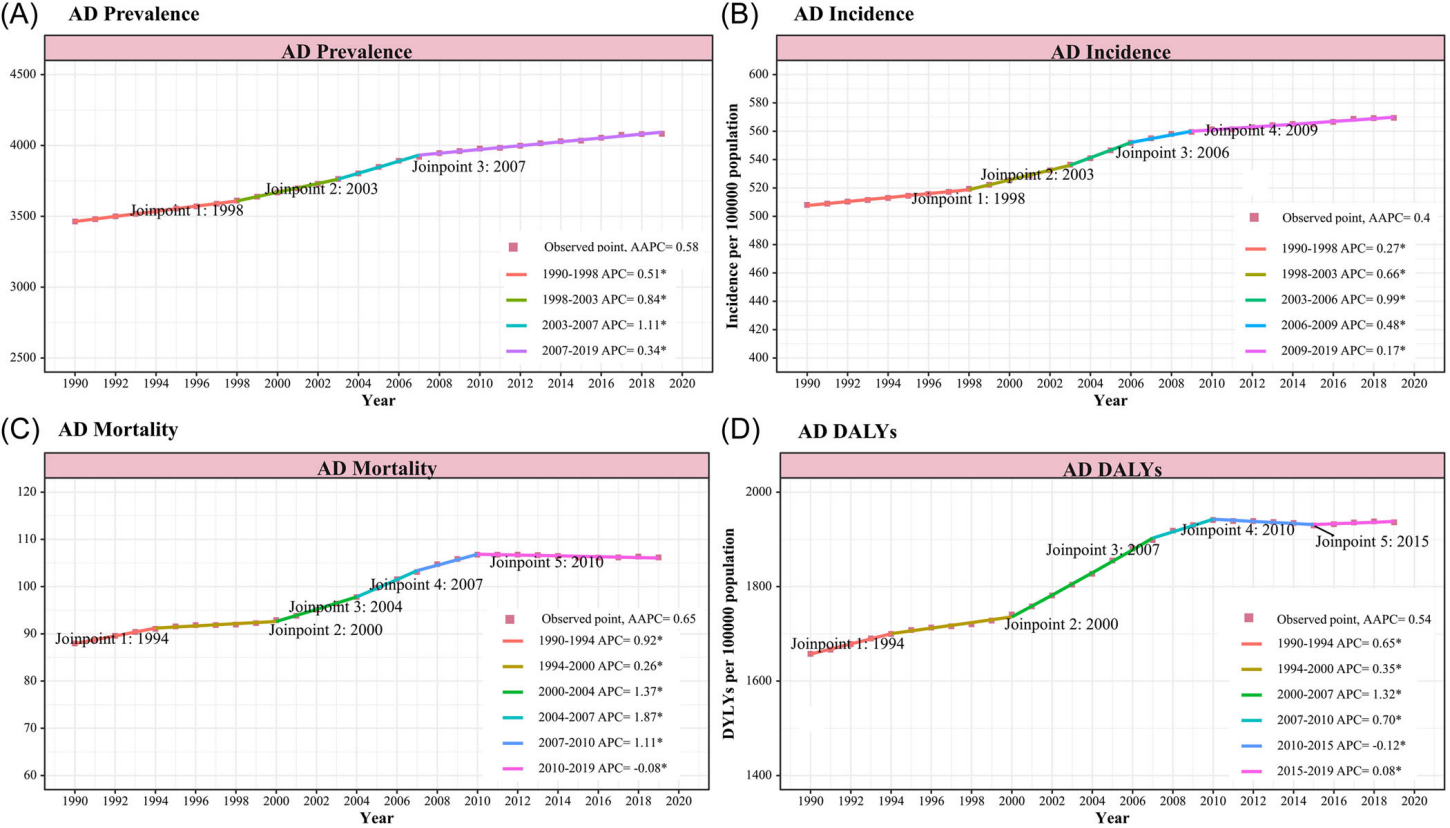
Joinpoint regression analysis of global Alzheimer's disease (AD) and other dementias. The prevalence (A), incidence (B), mortality (C), and DALYs (D) in older adults aged 60−89 years from 1990 to 2019 were described, respectively. APC, annual percentage change; DALYs, disability‐adjusted life years. [Color figure can be viewed at wileyonlinelibrary.com]

Globally, the incidence of AD and other dementias among older adults increased between 1990 and 1999 (AAPC: 0.31 [95% CI: 0.29 to 0.34]), reached a greater rate between 2000 and 2009 (AAPC: 0.71 [95% CI: 0.60 to 0.81]), and continued to increase between 2010 and 2019 (AAPC: 0.17 [95% CI: 0.16 to 0.19]) (Table 1). Overall, the incidence of AD and other dementias increased from 507.96 per 100,000 (95% UI: 420.39−604.50) in 1990 to 569.39 per 100,000 (95% UI: 476.88−667.35) in 2019 (AAPC: 0.40 [95% CI: 0.37 to 0.43]) (Table 2). The joinpoint regression analysis revealed that the prevalence of AD and other dementias was comparably stably increased with time, while a substantial change in the incidence of AD and other dementias occurred in 1998, 2003, 2006, and 2009 (Figure 1A,B). Notably, as for mortality, there are notable joinpoints in 2000, 2004, 2007, and 2010, reflecting an increasing trend. Specifically, the APC from 2000 to 2010 shows a consistent rise, with particularly high APC from 2000 to 2004 (APC: 1.37) and from 2004 to 2007 (APC: 1.87). This period signifies a phase of substantial increase in Alzheimer's mortality (Figure 1C). Similar to mortality, the DALYs show notable joinpoints in 2000, 2007, and 2010, marking significant growth in the burden of the disease. The APC during the period from 2000 to 2007 (APC: 1.32) and from 2007 to 2010 (APC: 0.70) demonstrate marked increases in AD's impact in terms of years of life lost or lived with disability (Figure 1D).

**Table 1:** Global AAPCs in prevalence, incidence, mortality, and DALYs of Alzheimer's disease and other dementias.

Period	Incidence		Prevalence		Mortality		DALYs	
	AAPC (95% CI)	*p* Value	AAPC (95% CI)	*p* Value	AAPC (95% CI)	*p* Value	AAPC (95% CI)	*p* Value
1990−1999	0.31 (0.29 to 0.34)	<0.001	0.55 (0.52 to 0.58)	<0.001	0.55 (0.48 to 0.62)	<0.001	0.48 (0.44 to 0.52)	<0.001
2000−2009	0.71 (0.60 to 0.81)	<0.001	0.85 (0.78 to 0.92)	<0.001	1.48 (1.31 to 1.64)	<0.001	1.18 (1.12 to 1.24)	<0.001
2010−2019	0.17 (0.16 to 0.19)	<0.001	0.34 (0.32 to 0.35)	<0.001	−0.08 (−0.12 to −0.05)	<0.001	−0.03 (−0.08 to 0.02)	0.296
1990−2019	0.40 (0.37 to 0.43)	<0.001	0.58 (0.55 to 0.60)	<0.001	0.65 (0.59 to 0.71)	<0.001	0.54 (0.51 to 0.57)	<0.001

Abbreviations: AAPC, average annual percentage change; DALYs, disability‐adjusted life years.

**Table 2:** The incidence and DALYs of Alzheimer's disease and other dementias and their AAPCs between sexes, ages, and locations from 1990 to 2019.

Factors	Incidence						DALYs					
	Cases (*n*), 1990	Incidence (per 100,000 population), 1990	Cases (*n*), 2019	Incidence (per 100,000 population), 2019	AAPC, 1990−2019	*p* Value	Cases (*n*), 1990	DALYs (per 100,000 population), 1990	Cases (*n*), 2019	DALYs (per 100,000 population), 2019	AAPC, 1990−2019	*p* Value
Global	2,439,455 (2,018,882 to 2,903,065)	507.96 (420.39 to 604.50)	5,768,862 (4,831,568 to 6,761,381)	569.39 (476.88 to 667.35)	0.40 (0.37 to 0.43)	<0.001	7,959,663 (3,513,415 to 17,995,886)	1657.43 (731.59 to 3747.25)	19,614,078 (8,777,973 to 43,065,827)	1935.93 (866.39 to 4250.63)	0.54 (0.51 to 0.57)	<0.001
Sex												
Male	839,360 (687,748 to 999,440)	387.56 (317.56 to 461.48)	2,185,302 (1,808,776 to 2,578,839)	462.40 (382.73 to 545.67)	0.61 (0.58 to 0.65)	<0.001	2,836,426 (1,220,832 to 6,580,085)	1309.68 (563.70 to 3038.25)	7,620,044 (3,322,975 to 17,407,790)	1612.36 (703.12 to 3683.41)	0.72 (0.69 to 0.76)	<0.001
Female	1,600,095 (1,333,051 to 1,894,032)	606.86 (505.58 to 718.34)	3,583,560 (3,006,303 to 4,202,105)	662.93 (556.14 to 777.36)	0.31 (0.26 to 0.36)	<0.001	5,123,237 (2,275,139 to 11,574,233)	1943.06 (862.88 to 4389.70)	11,994,035 (5,466,610 to 25,879,330)	2218.81 (1011.28 to 4787.49)	0.46 (0.42 to 0.49)	<0.001
Age group (years)												
60−64	177,936 (118,380 to 256,149)	110.76 (73.69 to 159.45)	396,898 (268,835 to 561,987)	126.99 (86.02 to 179.82)	0.47 (0.44 to 0.51)	<0.001	545,599 (214,118 to 1,317,922)	339.63 (133.29 to 820.40)	1,138,842 (455,698 to 2,708,611)	364.39 (145.81 to 866.66)	0.25 (0.19 to 0.31)	<0.001
65−69	271,802 (183,612 to 394,598)	220.10 (148.69 to 319.54)	637,907 (433,843 to 914,136)	246.69 (167.78 to 353.52)	0.39 (0.38 to 0.41)	<0.001	808,231 (322,548 to 1,969,272)	654.51 (261.21 to 1594.70)	1,821,266 (742,846 to 4,327,182)	704.32 (287.27 to 1673.42)	0.26 (0.21 to 0.32)	<0.001
60−79	1,422,559 (1,132,367 to 1,755,814)	330.86 (263.37 to 408.37)	320,7291 (2,576,686 to 3,929,971)	362.30 (291.07 to 443.93)	0.31 (0.28 to 0.34)	<0.001	4,038,555 (1,784,266 to 9,309,249)	939.30 (414.99 to 2165.17)	9,050,103 (4,069,636 to 20,596,722)	1022.31 (459.71 to 2326.63)	0.30 (0.27 to 0.33)	<0.001
60−89	2,439,455 (2,018,882 to 2,903,065)	507.96 (420.39 to 604.50)	5,768,862 (4,831,568 to 6,761,381)	569.39 (476.88 to 667.35)	0.40 (0.37 to 0.43)	<0.001	7,959,663 (3,513,415 to 17,995,886)	1657.43 (731.59 to 3747.25)	19,614,078 (8,777,973 to 43,065,827)	1935.93 (866.39 to 4250.63)	0.54 (0.51 to 0.57)	<0.001
Sociodemographic index												
High‐middle	725,137 (593,780 to 863,938)	541.09 (443.07 to 644.66)	1,643,347 (1,358,084 to 1,946,390)	634.41 (524.28 to 751.42)	0.56 (0.49 to 0.63)	<0.001	2,277,940 (1,030,561 to 5,115,134)	1699.76 (768.99 to 3816.83)	5,372,620 (2,478,013 to 11,696,968)	2074.08 (956.63 to 4515.57)	0.69 (0.62 to 0.75)	<0.001
High	874,875 (725,256 to 1,032,223)	656.68 (544.38 to 774.79)	1,695,188 (1,432,944 to 1,972,452)	724.54 (612.45 to 843.04)	0.35 (0.30 to 0.40)	<0.001	2,661,814 (1,227,640 to 5,858,975)	1997.96 (921.47 to 4397.76)	5,369,120 (2,545,026 to 11,280,869)	2294.81 (1087.77 to 4821.54)	0.47 (0.41 to 0.52)	<0.001
Low‐middle	241,130 (200,306 to 285,460)	356.31 (295.99 to 421.82)	663,480 (550,207 to 788,127)	412.44 (342.02 to 489.92)	0.51 (0.47 to 0.54)	<0.001	848,827 (360,529 to 2,020,682)	1254.31 (532.75 to 2985.92)	2,519,146 (1,052,562 to 5,870,995)	1565.97 (654.33 to 3649.56)	0.78 (0.71 to 0.85)	<0.001
Low	92,988 (77,335 to 110,715)	351.37 (292.22 to 418.35)	217,573 (181,476 to 256,432)	381.39 (318.11 to 449.51)	0.29 (0.26 to 0.31)	<0.001	326,539 (137,534 to 777,439)	1233.87 (519.69 to 2937.66)	847,047 (350,441 to 2,027,747)	1484.82 (614.30 to 3554.50)	0.64 (0.62 to 0.67)	<0.001
Middle	504,109 (416,981 to 598,415)	424.99 (351.53 to 504.49)	1,546,674 (1,277,943 to 1,828,880)	512.58 (423.52 to 606.11)	0.64 (0.62 to 0.66)	<0.001	1,840,231 (770,417 to 4,308,022)	1551.40 (649.52 to 3631.85)	5,496,660 (2,386,945 to 12,456,748)	1821.64 (791.05 to 4128.27)	0.56 (0.52 to 0.60)	<0.001
Region												
East Asia	451,075 (369,001 to 540,910)	432.18 (353.54 to 518.25)	1,574,968 (1,291,216 to 1,869,964)	598.51 (490.68 to 710.61)	1.12 (1.08 to 1.15)	<0.001	1,633,339 (680,140 to 3,844,378)	1564.91 (651.64 to 3683.31)	5,208,997 (2,366,850 to 11,365,774)	1979.52 (899.44 to 4319.17)	0.82 (0.75 to 0.88)	<0.001
Oceania	1202 (992 to 1434)	367.67 (303.32 to 438.77)	2824 (2316 to 3342)	385.82 (316.41 to 456.49)	0.17 (0.08 to 0.26)	<0.001	4410 (1790 to 10,390)	1349.13 (547.55 to 3178.55)	10,040 (4197 to 23,640)	1371.50 (573.34 to 3229.32)	0.03 (−0.04 to 0.11)	<0.001
Central Asia	31,768 (26,169 to 37,649)	570.68 (470.09 to 676.32)	41,161 (34,023 to 48,715)	492.75 (407.30 to 583.18)	−0.48 (−0.64 to −0.33)	<0.001	96,606 (44,767 to 214,848)	1735.43 (804.20 to 3859.53)	125,282 (57,620 to 284,628)	1499.78 (689.78 to 3407.35)	−0.50 (−0.72 to −0.28)	<0.001
Central Europe	116,330 (94,058 to 139,581)	608.54 (492.03 to 730.17)	202,728 (165,402 to 241,193)	711.36 (580.38 to 846.33)	0.53 (0.45 to 0.61)	<0.001	361,350 (163,340 to 816,219)	1890.27 (854.46 to 4269.76)	630,096 (290,305 to 1,357,682)	2210.96 (1018.66 to 4764.02)	0.54 (0.46 to 0.63)	<0.001
Eastern Europe	202,336 (162,210 to 244,935)	556.08 (445.81 to 673.16)	293,495 (236,953 to 352,698)	643.62 (519.61 to 773.42)	0.56 (0.32 to 0.79)	<0.001	573,366 (265,655 to 1,284,481)	1575.80 (730.11 to 3530.17)	884,301 (408,938 to 1,951,148)	1939.16 (896.75 to 4278.62)	0.75 (0.55 to 0.95)	<0.001
High‐income Asia Pacific	141,954 (117,605 to 169,093)	565.63 (468.61 to 673.77)	478,789 (393,461 to 567,676)	890.01 (731.40 to 1055.24)	1.60 (1.45 to 1.75)	<0.001	445,392 (199,079 to 990,892)	1774.71 (793.25 to 3948.31)	1,637,470 (759,101 to 3,376,117)	3043.86 (1411.08 to 6275.88)	1.88 (1.76 to 1.99)	<0.001
Australasia	18,009 (14,962 to 21,169)	592.63 (492.37 to 696.62)	39,874 (32,976 to 46,905)	645.88 (534.15 to 759.78)	0.30 (0.26 to 0.34)	<0.001	57,141 (25,975 to 125,748)	1880.39 (854.79 to 4138.11)	131,794 (61,162 to 280,971)	2134.82 (990.71 to 4551.21)	0.45 (0.39 to 0.52)	<0.001
Western Europe	489,326 (412,039 to 571,549)	656.27 (552.61 to 766.54)	792,353 (651,600 to 941,976)	723.64 (595.09 to 860.29)	0.34 (0.27 to 0.41)	<0.001	1,491,330 (685,838 to 3,267,967)	2000.13 (919.82 to 4382.89)	2,563,523 (1,194,091 to 5,516,114)	2341.21 (1090.54 to 5037.75)	0.54 (0.49 to 0.60)	<0.001
Southern Latin America	31,514 (25,801 to 37,893)	541.15 (443.06 to 650.69)	65,247 (53,525 to 77,222)	625.69 (513.28 to 740.52)	0.50 (0.48 to 0.52)	<0.001	95,375 (44,159 to 213,420)	1637.76 (758.29 to 3664.79)	203,148 (93,669 to 445,337)	1948.09 (898.24 to 4270.57)	0.61 (0.56 to 0.65)	<0.001
High‐income North America	334,866 (274,701 to 397,663)	742.18 (608.83 to 881.36)	546,721 (475,697 to 614,263)	682.07 (593.46 to 766.33)	−0.28 (−0.33 to −0.23)	<0.001	947,524 (455,207 to 2,029,648)	2100.05 (1008.90 to 4498.41)	1,561,138 (765,563 to 3,291,118)	1947.62 (955.09 to 4105.88)	−0.26 (−0.36 to −0.16)	<0.001
Caribbean	14,419 (11,910 to 17,159)	454.53 (375.42 to 540.89)	29,729 (24,665 to 35,119)	475.75 (394.71 to 561.99)	0.16 (0.13 to 0.18)	<0.001	55,488 (23,226 to 129,379)	1749.12 (732.16 to 4078.37)	115,026 (48,897 to 260,434)	1840.73 (782.48 to 4167.65)	0.18 (0.11 to 0.25)	<0.001
Andean Latin America	10,475 (8641 to 12,403)	447.78 (369.36 to 530.20)	33,000 (27,164 to 38,946)	495.73 (408.06 to 585.05)	0.37 (0.29 to 0.45)	<0.001	40,435 (16,812 to 94,840)	1728.47 (718.65 to 4054.14)	125,279 (53,029 to 284,577)	1881.96 (796.61 to 4274.96)	0.30 (0.23 to 0.37)	<0.001
Central Latin America	43,627 (36,031 to 52,054)	457.46 (377.81 to 545.82)	135,833 (112,120 to 160,906)	482.23 (398.05 to 571.24)	0.18 (0.15 to 0.22)	<0.001	172,883 (70,469 to 410,265)	1812.78 (738.91 to 4301.89)	553,409 (229,217 to 1,269,229)	1964.74 (813.76 to 4505.99)	0.28 (0.23 to 0.33)	<0.001
Tropical Latin America	56,347 (47,020 to 66,560)	529.04 (441.46 to 624.93)	179,014 (150,010 to 208,901)	607.56 (509.12 to 708.99)	0.49 (0.47 to 0.51)	<0.001	205,455 (88,265 to 471,710)	1929.12 (828.72 to 4428.85)	679,943 (298,965 to 1,510,394)	2307.67 (1014.66 to 5126.15)	0.63 (0.54 to 0.71)	<0.001
North Africa and Middle East	106,777 (88,994 to 126,047)	544.21 (453.58 to 642.43)	290,619 (242,333 to 341,683)	598.95 (499.43 to 704.19)	0.34 (0.28 to 0.40)	<0.001	358,925 (156,358 to 826,700)	1829.34 (796.91 to 4213.45)	972,474 (429,078 to 2,172,027)	2004.21 (884.34 to 4476.41)	0.32 (0.26 to 0.38)	<0.001
South Asia	177,442 (147,025 to 210,443)	283.45 (234.86 to 336.17)	554,094 (460,317 to 658,009)	331.72 (275.56 to 393.91)	0.54 (0.49 to 0.60)	<0.001	612,060 (259,416 to 1,474,755)	977.72 (414.40 to 2355.82)	217,6371 (896,262 to 5,243,949)	1302.85 (536.53 to 3139.21)	1.00 (0.90 to 1.11)	<0.001
Central sub‐Saharan Africa	10,414 (8616 to 12,476)	411.88 (340.76 to 493.41)	26,571 (22,339 to 31,041)	480.32 (403.81 to 561.13)	0.54 (0.50 to 0.58)	<0.001	31,928 (14,091 to 74,318)	1262.73 (557.31 to 2939.23)	91,685 (40,169 to 212,706)	1657.44 (726.14 to 3845.07)	0.94 (0.89 to 1.00)	<0.001
Eastern sub‐Saharan Africa	32,862 (27,425 to 38,976)	392.58 (327.63 to 465.62)	73,197 (61,405 to 85,694)	419.14 (351.62 to 490.71)	0.23 (0.20 to 0.27)	<0.001	114,885 (48,651 to 269,652)	1372.46 (581.21 to 3221.36)	287,120 (118,928 to 684,526)	1644.09 (681.02 to 3919.70)	0.61 (0.56 to 0.67)	<0.001
Southern sub‐Saharan Africa	15,360 (12,788 to 18,011)	481.16 (400.62 to 564.21)	31,046 (25,851 to 36,622)	471.38 (392.50 to 556.03)	−0.07 (−0.11 to −0.04)	<0.001	50,929 (22,286 to 117,206)	1595.37 (698.13 to 3671.51)	105,649 (45,662 to 244,701)	1604.08 (693.29 to 3715.32)	0.03 (−0.01 to 0.07)	<0.001
Western sub‐Saharan Africa	35,037 (28,984 to 41,699)	349.71 (289.32 to 416.21)	70,264 (58,397 to 82,986)	349.25 (290.27 to 412.49)	−0.01 (−0.04 to 0.03)	<0.001	141,178 (56,551 to 342,213)	1409.13 (564.45 to 3415.72)	313,139 (122,014 to 748,565)	1556.51 (606.49 to 3720.84)	0.33 (0.24 to 0.41)	<0.001
Southeast Asia	118,316 (98,379 to 139,564)	410.77 (341.55 to 484.54)	307,333 (255,924 to 360,398)	433.02 (360.59 to 507.79)	0.19 (0.15 to 0.22)	<0.001	469,664 (193,126 to 1,097,782)	1630.58 (670.50 to 3811.28)	1,238,193 (508,217 to 2,798,372)	1744.58 (716.06 to 3942.83)	0.23 (0.20 to 0.27)	<0.001

*Note*: Data in parentheses are 95% uncertainty intervals for cases, incidence, and DALYs and 95% CIs for AAPCs.

Abbreviations: AAPC, average annual percentage change; DALYs, disability‐adjusted life years; UI, uncertainty interval.

### 3.2 Global trends stratified by sex

There were global increases in the incidences of AD and other dementias from 1990 to 2019 in males and females, respectively, with an AAPC of 0.61 (95% CI: 0.58 to 0.65; from 387.56 per 100,000 population [95% UI: 317.56−461.48] in 1990 to 462.40 per 100,000 [95% UI: 382.73−545.67]) in males and 0.31 (95% CI: 0.26 to 0.36; from 606.86 per 100,000 population [95% UI: 505.58−718.34] to 662.93 per 100,000 population [95% UI: 556.14−777.36]) in females (Table 2). In addition, both male and female populations had increased numbers of DALYs due to AD and other dementias in the past three decades (Table 2). The AAPC of DALYs was 0.72 (95% CI: 0.69 to 0.76) in males and 0.46 (95% CI: 0.42 to 0.49) in females. A total of 5.8 million global incident AD and other dementia cases were recorded in 2019, among which 3.6 million (62.11%) occurred in females.

### 3.3 Global trends stratified by age

Globally, the greatest increase in the incidence of AD and other dementias between 1990 and 2019 was observed in individuals aged between 60 and 64 years (from 110.76 per 100,000 population [95% UI: 73.69−159.45] in 1990 to 126.99 per 100,000 [95% UI: 86.02−179.82] in 2019; AAPC: 0.47 [95% CI: 0.44 to 0.51]). We also observed a similar trend in individuals aged between 65 and 69 years (from 220.10 per 100,000 population [95% UI: 148.69−319.54] in 1990 to 246.69 per 100,000 [95% UI: 167.78−353.52] in 2019; AAPC: 0.39 [95% CI: 0.38 to 0.41]). For those aged 60 and 79 years, the incidence rose from 330.86 per 100,000 population [95% UI: 263.37−408.37] in 1990 to 362.30 per 100,000 [95% UI: 291.07−443.93] in 2019, with the AAPC of 0.31 [95% CI: 0.28 to 0.34]. Additionally, individuals aged 60−89 years experienced an increase from 507.96 per 100,000 population [95% UI: 420.39−604.50] in 1990 to 569.39 per 100,000 [95% UI: 476.88−667.35] in 2019, with the AAPC of 0.40 [95% CI: 0.37 to 0.43] during the same periods (Table 2). The greatest increase of DALYs due to AD and other dementias during 1990 and 2019 was observed in those aged 60 and 89 years (from 1657.43 per 100,000 population [95% UI: 731.59−3747.25] in 1990 to 1935.93 per 100,000 [95% UI: 866.39−4250.63] in 2019; AAPC: 0.54 [95% CI: 0.51 to 0.57]). Another three age groups also showed decreasing DALYs from AD and other dementias between 1990 and 2019 (Table 2). In 2019, 5,768,862 patients aged 60 and 89 years were diagnosed with incident AD and other dementias (Table 2).

### 3.4 Global trends stratified by SDI

The greatest increase in the incidence of AD and other dementias according to the SDI quintile was found in the middle‐SDI quintile (from 424.99 per 100,000 population [95% UI: 351.53−504.49] in 1990 to 512.58 per 100,000 [95% UI: 423.52−606.11]; AAPC: 0.64 [95% CI: 0.62 to 0.66]). Other SDI quintiles, such as high‐middle, high, low‐middle, and low‐SDI countries, also showed substantial increasing trends in the incidence of AD and other dementias during this time period. In 2019, high‐SDI quintile countries had the highest incidence (724.54 per 100,000 [95% UI: 612.45−843.04]) and low‐SDI quintile countries had the lowest incidence (381.39, 95% UI: 318.11−449.51) (Table 2).

### 3.5 Regional trends

At the regional level, the greatest increases in incidences of AD and other dementias during 1990 and 2019 were observed in East Asia (from 432.18 per 100,000 population [95% UI: 353.54−518.25] in 1990 to 598.51 per 100,000 [95% UI: 490.68−710.61] in 2019; AAPC: 1.12 [95% CI: 1.08 to 1.15]) and high‐income Asia Pacific region (from 565.63 per 100,000 population [95% UI: 468.61−673.77] in 1990 to 890.01 per 100,000 [95% UI: 731.40−1055.24] in 2019; AAPC: 1.60 [95% CI: 1.45 to 1.75]). Specifically, in 2019, high‐income Asia Pacific region had the highest incidence of AD and other dementias (890.01 per 100,000 population [95% UI: 731.40−1055.24]) (Table 2).

At the regional level during 1999 and 2019, high‐income Asian Pacific (from 1774.71 per 100,000 population [95% UI: 793.25−3948.30] in 1990 to 3043.86 per 100,000 [95% UI: 1411.08−6275.80] in 2019; AAPC: 1.88 [95% CI: 1.76 to 1.99]) and South Asia (from 977.72 per 100,000 population [95% UI: 414.40−2355.82] in 1990 to 1302.85 per 100,000 [95% UI: 536.53−3139.21]; AAPC: 1.00 [95% CI: 0.90 to 1.11]) had the highest DALYs increasing rate. Typically, in 2019, the high‐income Asia Pacific region had the highest DALYs due to AD and other dementias (Table 2).

### 3.6 National trends

At the national level, the most pronounced increase in the incidence of AD and other dementias between 1990 and 2019 occurred in Japan (from 572.54 per 100,000 population [95% UI: 471.73–684.72] to 977.47 per 100,000 population [95% UI: 797.41–1158.80]; AAPC: 1.88 [95% CI: 1.71 to 2.05]) and Iran (from 445.65 per 100,000 population [95% UI: 372.03–530.27] to 646.54 per 100,000 population [533.18–760.45]; AAPC: 1.31 [95% CI: 1.20 to 1.42]) (Supporting Information S1: Table S1). Specifically, the country with the highest incidence of AD and other dementias in 2019 was Japan (977.47 per 100,000 population [95% UI: 797.41–1158.80]).

During 1990 and 2019, the largest growth in DALYs from AD and other dementias was observed in Japan (from 1806.09 per 100,000 population [95% UI: 812.79–4017.46] to 3358.04 per 100,000 population [95% UI: 1565.40−6877.07]; AAPC: 2.15 [95% CI: 2.0 to –2.30]) (Supporting Information S1: Table S1). Specifically, the country with the highest DALYs from AD and other dementias was also Japan in 2019.

**Figure 2:**
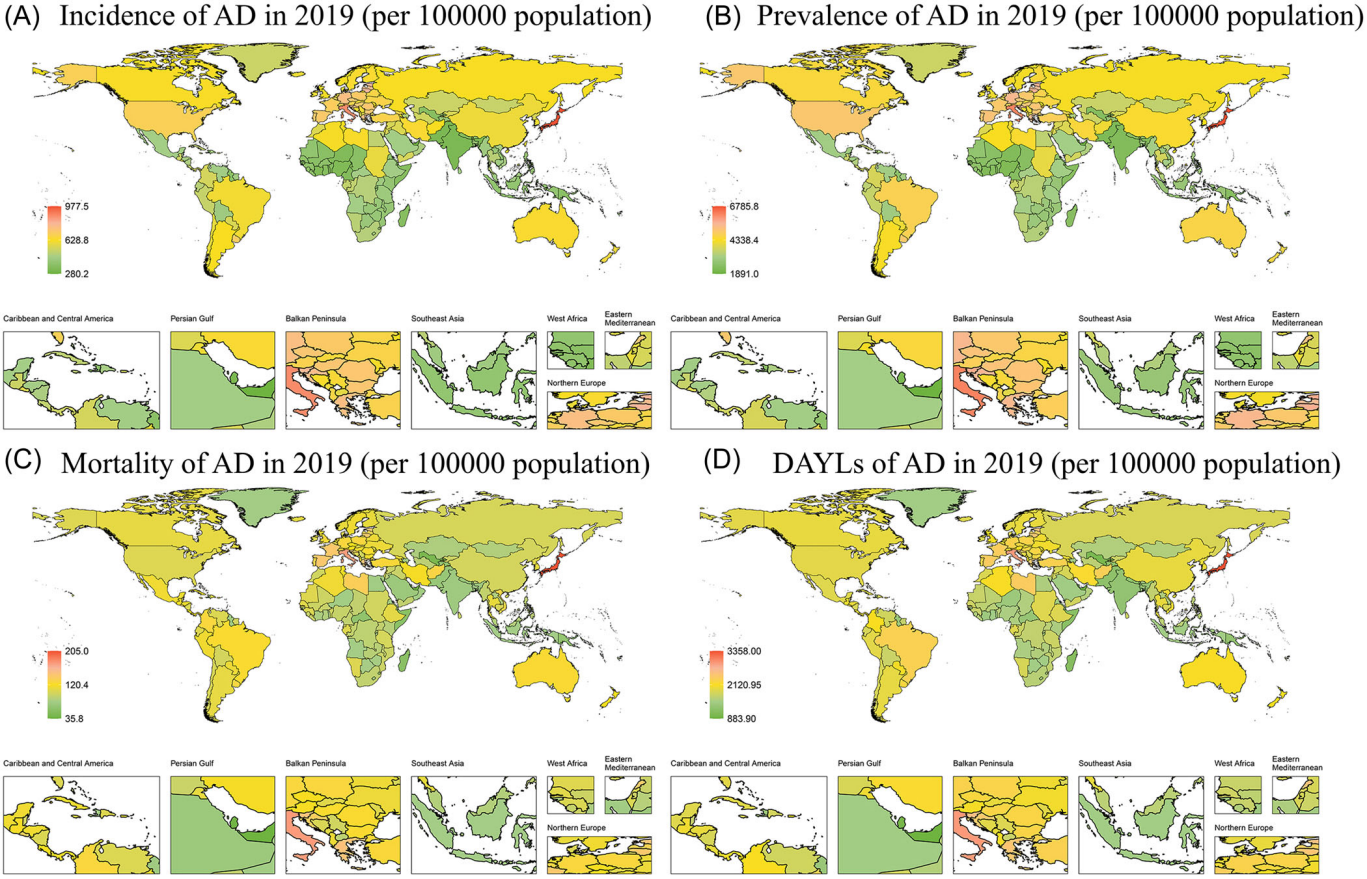
Global trend of incidence, prevalence, mortality, and DALYs of Alzheimer's disease (AD) and other dementias in 2019. The incidence (A), prevalence (B), mortality (C), and DALYs (D) of AD and other dementias in older adults aged 60−89 years in 2019 are shown, respectively. DALYs, disability‐adjusted life years. [Color figure can be viewed at wileyonlinelibrary.com]

It is worth noting that Japan shared the highest incidence, prevalence, mortality, and DALYs in 2019, indicating that AD and other dementias have gradually become a heavy burden on Japan (Figure 2A−D).

## 4 DISCUSSION

To our knowledge, this is the first study to provide a comprehensive description of the incidence, prevalence, mortality, and DALYs of AD and other dementias in older people aged 60−89 years from 1999 to 2019. This study disclosed the current burden of AD and other dementias and the differences between countries and regions, which is beneficial to developing public strategies.

As the population ages, the number of people with dementia has increased worldwide in recent decades. Globally, the incidence of AD and other dementias among older adults increased between 1990 and 1999, reached a greater increasing rate between 2000 and 2009, and continuously increased between 2010 and 2019. By 2050, it is predicted that there will be 152.8 million cases of dementia worldwide.^14^ The proportion of the population over the age of 60 years is projected to increase by 22% by 2050, with this age group experiencing the fastest population growth.^15^ The combination of population growth and an aging population is leading to a higher prevalence of dementia, which in turn will drive up future medical costs associated with AD and other dementias. As per the WHO, dementia has emerged as the seventh most prevalent cause of mortality on a global scale.^16^

Both males and females experienced an increase in the number of years lived with disability due to AD and other dementias in the past three decades. Out of the 5.8 million global incident cases of AD and other dementias in 2019, 3.6 million (62.1%) occurred in females. During this period, DALYs (per 100,000 population) were consistently greater among women than among men. This indicates a greater disease burden on women. The differences in prevalence patterns between the sexes may be attributed to factors such as reproductive capacity, hormone levels, genetic susceptibility, and mental health.^17^ Women are more likely to develop structural and functional disorders of the nervous system and are twice as likely as men to experience psychological issues such as depression,^18^, ^19^ which act as risk factors for AD. Additionally, female brains are inherently more susceptible to AD due to the influence of sex hormones. Furthermore, because women generally live longer than men, they constitute a significant proportion of the aging population.^20^ Our research found that the disease burden in men is increasing more rapidly than that in women. This may be attributed to higher rates of smoking and drinking among men, which could explain this trend.^21^

Dementia, an age‐related disease, has seen a significant increase in incidence globally due to rising life expectancy.^22^ Globally, the most significant increase in the incidence of AD and other dementias between 1990 and 2019 was observed among individuals aged 60−64 years. Similarly, the greatest increase in DALYs caused by AD and other dementias during this period was observed among those aged 60−89 years.

The middle‐SDI quintile experienced the greatest increase in the incidence of AD and other dementias. In 2019, countries in the high‐SDI quintile had the highest incidence rates, while those in the low‐SDI quintile had the lowest incidence rates. This phenomenon may be attributed to the higher life expectancy in regions with high SDI scores, as well as the increasing proportion of elderly individuals in the population over time.^23^ Nevertheless, regions with higher SDIs have more potential to control these conditions since they enjoy improved healthcare services and allocate more funds for the treatment of individuals with dementia. Early identification of risk factors associated with dementia significantly decreases mortality rates and overall burden. Furthermore, high‐SDI regions prioritize investments in education and social welfare, contributing to enhanced physical, mental, and cognitive well‐being, thereby effectively managing risk factors for dementia at an earlier phase.^24^

Regionally, the greatest increase in the incidence of AD and other dementias between 1990 and 2019 was observed in East Asia and the high‐income Asia Pacific region. In terms of DALYs associated with AD and other dementias, the regions with the highest increasing rate were the high‐income regions of the Asia Pacific and South Asia. On a national scale, Japan and Iran experienced the most significant rise in the prevalence of AD and other dementias from 1990 to 2019. Specifically, Japan witnessed the largest increase in the burden of DALYs associated with AD and other dementias during this period. Differences in economic levels, policies, and cultures among countries may be the underlying reasons for these results. However, it is challenging to provide a unified explanation for these findings.

Certain determinants are linked with the increasing incidence and cases of AD and other types of dementias, such as population aging, advancement in medical techniques, unhealthy lifestyles, public awareness improvement, and genetic and environmental exposure. In detail, aging society promotes the incidence of AD and other types of dementias, which is pronounced in those with chronic diseases (e.g., obesity, hypertension, and diabetes). Increased public awareness and advanced medicine are beneficial to the early detection of aging diseases. Additionally, genetic predispositions interacting with environmental factors can also contribute to the risk of developing AD and other dementias. In summary, we speculated that significant increases in incidence in specific years (e.g., 1998, 2003, 2006, and 2009) was due to advancements in diagnostic capabilities, changes in diagnostic criteria, increased awareness and reporting, and potential environmental, lifestyle, or demographic shifts.

The limitations of this article can be summarized as follows: First, dementia subtypes were not further classified. For instance, dementia can be categorized as vascular dementia or dementia with Lewy bodies, which have different disease burdens and risk factors. Unfortunately, GBD 2019 data stratified by pathological subtype are lacking at this time. Second, there have been updates in diagnostic criteria, biomarkers, medical records, and insurance codes for dementia globally over the past three decades. This could introduce heterogeneity and discrepancies between our analysis and the actual disease burden in the real world. As a result, it is crucial to interpret the results of our analysis with caution. Therefore, further investigations are necessary to validate and expand on our findings.

## 5 CONCLUSIONS

In conclusion, this study highlights the global epidemiological trends of dementia from 1990 to 2019, emphasizing its emergence as a significant public health issue due to the aging population. The risk of developing dementia increases with age, making it crucial to prioritize attention given to females and elderly individuals. It is clear that most countries and territories continue to face a substantial disease burden. Our findings associated with the trends of AD and other dementias might provide insights into the potential risk of increasing dementias on healthy lifespan at a population scale, which is significant for healthcare professionals and decision‐makers to inform the ongoing debate on the policy of AD and other dementias.

## AUTHOR CONTRIBUTIONS

Mengdan Su, Tianhong Wang, and Keyu Cao conceived and designed the study. Congcong Zou and Fei Liu contributed to the acquisition of data, analysis, and interpretation. Mengdan Su and Fei Liu drafted the manuscript and assisted in the preparation of the figures. All authors have read and approved the final version of the manuscript.

## CONFLICT OF INTEREST STATEMENT

Fei Liu is the editorial member of Ibrain and a coauthor of this article. She was excluded from editorial decision‐making related to the acceptance and publication of this article. Editorial decision‐making was handled independently by editor‐in‐chiefs to minimize bias. The remaining authors declare no conflict of interest.

## ETHICS STATEMENT

The institutional review board of the West China Hospital of Sichuan University determined that the study did not need approval because it used publicly available data. Informed consent was obtained from all subjects before attending the survey of Global Burden of Disease Study.

## Supporting information

Supporting information.

## Data Availability

The data sets used and/or analyzed during the current study are available from the corresponding author upon reasonable request.
