# Supplementary material for: Global, regional, and national burdens of Alzheimer's disease and other forms of dementia in the elderly population from 1999 to 2019: A trend analysis based on the Global Burden of Disease Study 2019
Source: Ibrain. 2024 Sep 22;10(4):488–99. doi: 10.1002/ibra.12181 (PMC11649385; doi:10.1002/ibra.12181)
Supplement: Supplementary file 1 — Supporting information. [file IBRA-10-488-s001.docx]

***Supplementary Files***

**Global, regional, and national burdens of Alzheimer’s disease and other dementias in the elderly population from 1999-2019: a trend analysis based on the Global Burden of Disease Study 2019**

**Table S1.** The AAPC values in 204 countries and territories.

| **Countries and territories** | **DALYs** | **Incidence** |
| --- | --- | --- |
| Afghanistan | 0.48 (0.41 to 0.55) | 0.52 (0.44 to 0.59) |
| Albania | 0.53 (0.44 to 0.62) | 0.54 (0.48 to 0.61) |
| Algeria | 0.65 (0.59 to 0.7) | 0.58 (0.55 to 0.61) |
| American Samoa | 0.31 (0.17 to 0.45) | 0.48 (0.41 to 0.54) |
| Andorra | 1.01 (0.83 to 1.19) | 0.79 (0.55 to 1.03) |
| Angola | 0.67 (0.58 to 0.75) | 0.23 (0.19 to 0.28) |
| Antigua and Barbuda | -0.82 (-0.88 to -0.77) | -0.76 (-0.78 to -0.74) |
| Argentina | 0.66 (0.6 to 0.71) | 0.51 (0.49 to 0.54) |
| Armenia | 1.02 (0.8 to 1.24) | 0.73 (0.53 to 0.94) |
| Australia | 0.48 (0.41 to 0.55) | 0.3 (0.26 to 0.35) |
| Austria | 0.23 (0.09 to 0.37) | 0.12 (0 to 0.25) |
| Azerbaijan | -0.63 (-0.84 to -0.41) | -0.63 (-0.88 to -0.37) |
| Bahamas | -0.26 (-0.38 to -0.15) | -0.21 (-0.27 to -0.16) |
| Bahrain | -0.78 (-0.99 to -0.56) | -0.49 (-0.65 to -0.33) |
| Bangladesh | 0.22 (0.08 to 0.35) | 0.15 (0.11 to 0.2) |
| Barbados | -0.45 (-0.5 to -0.4) | -0.53 (-0.56 to -0.5) |
| Belarus | 0.45 (0.12 to 0.78) | 0.35 (0.18 to 0.52) |
| Belgium | 0.31 (0.13 to 0.48) | 0.14 (0.09 to 0.19) |
| Belize | -0.24 (-0.37 to -0.11) | -0.3 (-0.32 to -0.27) |
| Benin | -0.16 (-0.36 to 0.05) | -0.27 (-0.43 to -0.11) |
| Bermuda | 0.46 (0.36 to 0.56) | 0.5 (0.45 to 0.55) |
| Bhutan | 1.34 (1.28 to 1.4) | 0.74 (0.72 to 0.77) |
| Bolivia (Plurinational State of) | 0.36 (0.33 to 0.39) | 0.23 (0.2 to 0.27) |
| Bosnia and Herzegovina | 0.58 (0.42 to 0.74) | 0.65 (0.49 to 0.8) |
| Botswana | 0.28 (0.2 to 0.37) | 0.04 (-0.05 to 0.12) |
| Brazil | 0.64 (0.55 to 0.72) | 0.5 (0.48 to 0.52) |
| Brunei Darussalam | -0.11 (-0.2 to -0.02) | -0.32 (-0.39 to -0.26) |
| Bulgaria | 1.19 (1.11 to 1.28) | 1.02 (0.97 to 1.07) |
| Burkina Faso | 0.17 (0.09 to 0.25) | 0.24 (0.07 to 0.41) |
| Burundi | -0.12 (-0.18 to -0.05) | -0.54 (-0.57 to -0.52) |
| Cabo Verde | 0.2 (0 to 0.39) | -0.19 (-0.27 to -0.1) |
| Cambodia | 0.64 (0.61 to 0.67) | 0.26 (0.23 to 0.3) |
| Cameroon | 0.15 (0.04 to 0.26) | 0.17 (0.06 to 0.28) |
| Canada | 0.1 (0.03 to 0.18) | -0.08 (-0.13 to -0.02) |
| Central African Republic | 0.29 (0.24 to 0.34) | 0.25 (0.22 to 0.29) |
| Chad | -0.16 (-0.23 to -0.08) | -0.31 (-0.48 to -0.14) |
| Chile | 0.49 (0.43 to 0.55) | 0.54 (0.52 to 0.56) |
| China | 0.81 (0.74 to 0.87) | 1.12 (1.08 to 1.16) |
| Colombia | 0.7 (0.64 to 0.76) | 0.67 (0.61 to 0.74) |
| Comoros | 0.74 (0.63 to 0.86) | 0.2 (0.11 to 0.29) |
| Congo | 0.78 (0.68 to 0.88) | 0.46 (0.39 to 0.53) |
| Cook Islands | 0.09 (-0.16 to 0.34) | 0.57 (0.28 to 0.87) |
| Costa Rica | 0.06 (-0.04 to 0.16) | 0.07 (0.05 to 0.09) |
| Cte d'Ivoire | 0.52 (0.42 to 0.61) | 0.47 (0.42 to 0.52) |
| Croatia | 0.91 (0.77 to 1.06) | 0.83 (0.66 to 0.99) |
| Cuba | 0.18 (0.04 to 0.32) | 0.18 (0.14 to 0.21) |
| Cyprus | 0.46 (0.36 to 0.55) | 0.48 (0.42 to 0.55) |
| Czechia | 0.45 (0.29 to 0.6) | 0.44 (0.38 to 0.5) |
| Democratic People's Republic of Korea | 0.72 (0.58 to 0.86) | 0.77 (0.61 to 0.93) |
| Democratic Republic of the Congo | 1.09 (1.01 to 1.17) | 0.68 (0.61 to 0.75) |
| Denmark | -0.21 (-0.31 to -0.12) | -0.34 (-0.4 to -0.29) |
| Djibouti | 0.58 (0.48 to 0.68) | 0.13 (0.09 to 0.16) |
| Dominica | -0.18 (-0.25 to -0.1) | -0.16 (-0.21 to -0.11) |
| Dominican Republic | 0.32 (0.22 to 0.42) | 0.26 (0.21 to 0.32) |
| Ecuador | 0.05 (-0.1 to 0.21) | 0.16 (0.04 to 0.27) |
| Egypt | -0.24 (-0.33 to -0.15) | -0.13 (-0.21 to -0.06) |
| El Salvador | 0.54 (0.43 to 0.66) | 0.52 (0.5 to 0.54) |
| Equatorial Guinea | 1.09 (0.92 to 1.25) | 0.48 (0.42 to 0.55) |
| Eritrea | 1.34 (1.16 to 1.53) | 0.24 (0.05 to 0.43) |
| Estonia | 1.28 (1.1 to 1.46) | 0.95 (0.81 to 1.09) |
| Eswatini | -0.22 (-0.28 to -0.16) | -0.15 (-0.2 to -0.1) |
| Ethiopia | 1.46 (1.41 to 1.51) | 0.83 (0.77 to 0.89) |
| Fiji | -0.38 (-0.47 to -0.28) | -0.05 (-0.11 to 0.01) |
| Finland | 0.17 (0.12 to 0.22) | 0.06 (-0.05 to 0.18) |
| France | 0.48 (0.42 to 0.54) | 0.53 (0.37 to 0.69) |
| Gabon | 0.29 (0.2 to 0.38) | 0.14 (0.09 to 0.2) |
| Gambia | 0.87 (0.76 to 0.99) | 0.54 (0.5 to 0.59) |
| Georgia | 0.89 (0.71 to 1.07) | 0.66 (0.56 to 0.77) |
| Germany | 0.11 (-0.01 to 0.23) | 0.2 (0.14 to 0.27) |
| Ghana | 0.4 (0.34 to 0.46) | 0.34 (0.3 to 0.38) |
| Greece | 1.18 (1.11 to 1.26) | 0.91 (0.82 to 1) |
| Greenland | 0.24 (-0.05 to 0.53) | 0.24 (0 to 0.48) |
| Grenada | -1.1 (-1.19 to -1.01) | -1.01 (-1.07 to -0.95) |
| Guam | 0.72 (0.66 to 0.78) | 1.01 (0.97 to 1.04) |
| Guatemala | 1.07 (0.88 to 1.26) | 1.1 (1.02 to 1.18) |
| Guinea | 0.34 (0.27 to 0.4) | 0.18 (0.15 to 0.21) |
| Guinea-Bissau | 0.19 (0.13 to 0.25) | 0.11 (0.06 to 0.16) |
| Guyana | -0.22 (-0.34 to -0.11) | -0.16 (-0.28 to -0.04) |
| Haiti | 0.51 (0.46 to 0.55) | 0.24 (0.17 to 0.31) |
| Honduras | 0.26 (0.21 to 0.31) | -0.02 (-0.09 to 0.05) |
| Hungary | 0.63 (0.52 to 0.74) | 0.54 (0.45 to 0.64) |
| Iceland | 0.02 (-0.07 to 0.1) | -0.05 (-0.1 to 0) |
| India | 1.32 (1.16 to 1.48) | 0.76 (0.68 to 0.84) |
| Indonesia | 0.36 (0.27 to 0.45) | 0.08 (0.03 to 0.14) |
| Iran (Islamic Republic of) | 1.52 (1.4 to 1.64) | 1.31 (1.20 to 1.42) |
| Iraq | -0.33 (-0.4 to -0.27) | -0.26 (-0.31 to -0.22) |
| Ireland | 0.22 (0.16 to 0.29) | 0.07 (0.03 to 0.11) |
| Israel | 0.32 (0.24 to 0.41) | 0.15 (0.06 to 0.24) |
| Italy | 1.96 (1.83 to 2.08) | 0.89 (0.74 to 1.04) |
| Jamaica | -0.02 (-0.14 to 0.09) | -0.11 (-0.16 to -0.06) |
| Japan | 2.15 (2.01 to 2.30) | 1.88 (1.71 to 2.05) |
| Jordan | -0.01 (-0.08 to 0.07) | 0.24 (0.19 to 0.28) |
| Kazakhstan | -0.03 (-0.28 to 0.21) | -0.05 (-0.25 to 0.15) |
| Kenya | -0.07 (-0.12 to -0.03) | -0.21 (-0.24 to -0.18) |
| Kiribati | 0.1 (0.04 to 0.17) | -0.1 (-0.15 to -0.06) |
| Kuwait | 0.39 (-0.15 to 0.94) | 0.45 (-0.03 to 0.93) |
| Kyrgyzstan | -0.13 (-0.39 to 0.13) | -0.24 (-0.5 to 0.01) |
| Lao People's Democratic Republic | 0.46 (0.4 to 0.53) | 0.26 (0.2 to 0.33) |
| Latvia | 0.97 (0.88 to 1.05) | 0.82 (0.73 to 0.92) |
| Lebanon | 0.94 (0.86 to 1.01) | 1.01 (0.96 to 1.06) |
| Lesotho | -0.22 (-0.25 to -0.18) | -0.26 (-0.29 to -0.23) |
| Liberia | 0.18 (0.06 to 0.3) | 0.26 (0.14 to 0.37) |
| Libya | 0.1 (0.02 to 0.18) | 0.18 (0.11 to 0.25) |
| Lithuania | 0.83 (0.76 to 0.91) | 0.75 (0.6 to 0.9) |
| Luxembourg | 0.04 (-0.09 to 0.17) | -0.26 (-0.34 to -0.18) |
| Madagascar | -0.39 (-0.45 to -0.33) | -0.4 (-0.42 to -0.37) |
| Malawi | 0.76 (0.68 to 0.83) | 0.39 (0.33 to 0.45) |
| Malaysia | -0.22 (-0.35 to -0.09) | -0.13 (-0.22 to -0.05) |
| Maldives | 1.43 (1.27 to 1.58) | 1.28 (1.18 to 1.38) |
| Mali | 0.53 (0.44 to 0.63) | 0.36 (0.33 to 0.39) |
| Malta | 0.43 (0.31 to 0.55) | 0.31 (0.21 to 0.41) |
| Marshall Islands | -1.01 (-1.1 to -0.92) | -0.88 (-0.96 to -0.8) |
| Mauritania | 0.09 (-0.16 to 0.34) | 0.12 (-0.04 to 0.28) |
| Mauritius | 0.28 (0.1 to 0.46) | 0.24 (0.16 to 0.31) |
| Mexico | 0.12 (0.04 to 0.2) | -0.04 (-0.14 to 0.06) |
| Micronesia (Federated States of) | -0.3 (-0.36 to -0.23) | -0.26 (-0.32 to -0.19) |
| Monaco | -0.09 (-0.19 to 0) | -0.19 (-0.27 to -0.11) |
| Mongolia | -0.42 (-0.55 to -0.29) | -0.3 (-0.44 to -0.17) |
| Montenegro | 0.15 (-0.01 to 0.31) | 0.16 (0.02 to 0.3) |
| Morocco | 0.12 (0.02 to 0.23) | 0.05 (-0.01 to 0.12) |
| Mozambique | 0.58 (0.49 to 0.66) | 0.12 (0.08 to 0.16) |
| Myanmar | 0.61 (0.54 to 0.68) | 0.35 (0.3 to 0.4) |
| Namibia | 0.97 (0.87 to 1.06) | 0.51 (0.44 to 0.58) |
| Nauru | -1.36 (-1.51 to -1.21) | -0.95 (-1.07 to -0.84) |
| Nepal | 0.99 (0.97 to 1.01) | 0.39 (0.37 to 0.41) |
| Netherlands | 0.16 (0.07 to 0.25) | 0.04 (-0.04 to 0.11) |
| New Zealand | 0.3 (0.25 to 0.35) | 0.27 (0.21 to 0.33) |
| Nicaragua | 0.09 (-0.05 to 0.24) | 0.07 (0.03 to 0.11) |
| Niger | 0.39 (0.24 to 0.53) | 0.19 (0.03 to 0.35) |
| Nigeria | 0.44 (0.31 to 0.58) | -0.22 (-0.33 to -0.1) |
| Niue | -0.99 (-1.09 to -0.89) | -0.55 (-0.68 to -0.42) |
| North Macedonia | 0.34 (0.27 to 0.41) | 0.3 (0.25 to 0.35) |
| Northern Mariana Islands | -0.29 (-0.8 to 0.22) | -0.1 (-0.69 to 0.48) |
| Norway | -0.41 (-0.47 to -0.36) | -0.37 (-0.43 to -0.31) |
| Oman | -0.36 (-0.67 to -0.06) | -0.12 (-0.5 to 0.26) |
| Pakistan | -0.38 (-0.42 to -0.33) | -0.54 (-0.58 to -0.5) |
| Palau | -0.7 (-0.85 to -0.56) | -0.46 (-0.54 to -0.38) |
| Palestine | -0.36 (-0.45 to -0.26) | -0.15 (-0.25 to -0.05) |
| Panama | 0.3 (0.22 to 0.38) | 0.3 (0.27 to 0.33) |
| Papua New Guinea | 0.1 (-0.02 to 0.22) | 0.17 (-0.02 to 0.36) |
| Paraguay | 0.14 (0.06 to 0.22) | 0.03 (0.02 to 0.05) |
| Peru | 0.41 (0.34 to 0.48) | 0.49 (0.47 to 0.51) |
| Philippines | -0.67 (-0.85 to -0.49) | -0.16 (-0.21 to -0.11) |
| Poland | 0.18 (0.13 to 0.24) | 0.28 (0.19 to 0.36) |
| Portugal | 1.23 (1.15 to 1.3) | 0.87 (0.83 to 0.92) |
| Puerto Rico | 0.38 (0.32 to 0.45) | 0.42 (0.38 to 0.46) |
| Qatar | -1.31 (-1.56 to -1.07) | -0.96 (-1.08 to -0.84) |
| Republic of Korea | 0.82 (0.69 to 0.94) | 0.46 (0.35 to 0.56) |
| Republic of Moldova | 0.83 (0.68 to 0.97) | 0.71 (0.59 to 0.82) |
| Romania | 0.97 (0.87 to 1.08) | 0.83 (0.78 to 0.88) |
| Russian Federation | 0.7 (0.5 to 0.9) | 0.53 (0.38 to 0.68) |
| Rwanda | 0.8 (0.41 to 1.2) | 0.19 (-0.14 to 0.53) |
| Saint Kitts and Nevis | -0.92 (-1.01 to -0.82) | -0.9 (-0.95 to -0.86) |
| Saint Lucia | 0.34 (0.26 to 0.42) | 0.26 (0.22 to 0.3) |
| Saint Vincent and the Grenadines | -0.2 (-0.3 to -0.1) | -0.09 (-0.12 to -0.06) |
| Samoa | 0.09 (0.03 to 0.16) | 0.25 (0.15 to 0.35) |
| San Marino | 0.77 (0.74 to 0.81) | 0.64 (0.61 to 0.67) |
| Sao Tome and Principe | 0.47 (0.42 to 0.52) | 0.24 (0.19 to 0.3) |
| Saudi Arabia | -0.97 (-1.12 to -0.82) | -0.81 (-0.89 to -0.72) |
| Senegal | 0.46 (0.15 to 0.78) | 0.22 (0.15 to 0.28) |
| Serbia | 0.49 (0.38 to 0.6) | 0.64 (0.57 to 0.72) |
| Seychelles | -0.53 (-0.61 to -0.44) | -0.42 (-0.48 to -0.36) |
| Sierra Leone | 0.08 (-0.12 to 0.28) | -0.04 (-0.21 to 0.12) |
| Singapore | 0.38 (0.11 to 0.64) | 0.42 (0.32 to 0.52) |
| Slovakia | 0.03 (-0.08 to 0.14) | 0.15 (0.07 to 0.22) |
| Slovenia | 0.64 (0.5 to 0.79) | 0.56 (0.49 to 0.63) |
| Solomon Islands | 0.62 (0.57 to 0.68) | 0.52 (0.47 to 0.57) |
| Somalia | -0.12 (-0.2 to -0.04) | -0.28 (-0.32 to -0.24) |
| South Africa | -0.03 (-0.12 to 0.06) | -0.12 (-0.17 to -0.08) |
| South Sudan | -0.1 (-0.16 to -0.05) | -0.22 (-0.27 to -0.17) |
| Spain | 0.3 (0.11 to 0.49) | 0.21 (0.08 to 0.33) |
| Sri Lanka | 0.03 (-0.08 to 0.15) | 0.23 (0.18 to 0.27) |
| Sudan | 0.07 (0.03 to 0.1) | 0.1 (0.06 to 0.14) |
| Suriname | 0.05 (-0.03 to 0.12) | 0.06 (0.02 to 0.1) |
| Sweden | -0.4 (-0.49 to -0.31) | -0.4 (-0.47 to -0.33) |
| Switzerland | 0.15 (0.1 to 0.19) | 0.03 (0 to 0.07) |
| Syrian Arab Republic | 0.03 (-0.17 to 0.23) | 0.01 (-0.09 to 0.1) |
| Taiwan (Province of China) | 1.39 (1.29 to 1.48) | 1.3 (1.27 to 1.34) |
| Tajikistan | -1.53 (-1.79 to -1.27) | -1.41 (-1.63 to -1.19) |
| Thailand | 0.37 (0.27 to 0.47) | 0.67 (0.64 to 0.71) |
| Timor-Leste | 0.38 (0.02 to 0.75) | 0.2 (-0.16 to 0.57) |
| Togo | -0.13 (-0.29 to 0.03) | -0.05 (-0.2 to 0.09) |
| Tokelau | -0.68 (-0.87 to -0.49) | -0.29 (-0.46 to -0.11) |
| Tonga | 0.72 (0.65 to 0.8) | 0.75 (0.71 to 0.79) |
| Trinidad and Tobago | -0.17 (-0.26 to -0.07) | -0.13 (-0.17 to -0.08) |
| Tunisia | 0.4 (0.35 to 0.44) | 0.38 (0.33 to 0.43) |
| Turkey | 0.28 (0.17 to 0.38) | 0.36 (0.25 to 0.48) |
| Turkmenistan | -0.01 (-0.11 to 0.09) | 0.01 (-0.1 to 0.12) |
| Tuvalu | 0.18 (0.11 to 0.24) | 0.45 (0.37 to 0.53) |
| Uganda | 0.46 (0.35 to 0.58) | 0.13 (0.06 to 0.2) |
| Ukraine | 0.97 (0.79 to 1.16) | 0.62 (0.41 to 0.82) |
| United Arab Emirates | -1.49 (-1.63 to -1.35) | -1.28 (-1.41 to -1.15) |
| United Kingdom | 0.27 (0.19 to 0.34) | 0.11 (0.05 to 0.17) |
| United Republic of Tanzania | 0.62 (0.53 to 0.71) | 0.29 (0.22 to 0.36) |
| United States of America | -0.3 (-0.4 to -0.2) | -0.3 (-0.35 to -0.24) |
| United States Virgin Islands | -0.06 (-0.13 to 0.01) | 0.19 (0.16 to 0.23) |
| Uruguay | 0.72 (0.63 to 0.81) | 0.62 (0.6 to 0.64) |
| Uzbekistan | -2.07 (-2.16 to -1.97) | -1.71 (-1.81 to -1.61) |
| Vanuatu | 0.13 (-0.12 to 0.39) | 0.13 (0.06 to 0.2) |
| Venezuela (Bolivarian Republic of) | -0.12 (-0.23 to -0.02) | -0.19 (-0.24 to -0.14) |
| Viet Nam | 0.34 (0.25 to 0.43) | 0.06 (-0.02 to 0.13) |
| Yemen | 0.37 (0.32 to 0.42) | 0.23 (0.16 to 0.3) |
| Zambia | 0.39 (0.31 to 0.47) | 0.24 (0.2 to 0.28) |
| Zimbabwe | 0.03 (-0.01 to 0.08) | -0.03 (-0.09 to 0.03) |

Note. DALYs: Disability-Adjusted Life Years; AAPC: average annual percentage changes.
